# Methamphetamine-induced psychosis: Clinical features, treatment modalities and outcomes

**DOI:** 10.4102/sajpsychiatry.v22i1.980

**Published:** 2016-09-29

**Authors:** Eileen Thomas, Helena Lategan, Chris Verster, Martin Kidd, Lize Weich

**Affiliations:** 1 Department of Psychiatry, Stellenbosch University, South Africa; 2 Centre for Statistical Consultation, Stellenbosch University, South Africa

## Abstract

**Objective:**

To investigate the clinical features, prescribing patterns and outcomes of psychiatric inpatients admitted with methamphetamine-induced psychosis.

**Method:**

A cross-sectional, descriptive pilot study was conducted between March 2014 and August 2014 at three South African Mental Health Care Act designated hospitals prior to admission to a psychiatric hospital. Patients with methamphetamine-related psychotic symptoms according to the DSM-5 criteria were eligible. Structured face-to-face interviews were conducted and the Brief Psychiatric Rating Scale was employed as a measure of current psychopathology.

**Results:**

Fifty-six participants were included. Positive psychotic symptoms (e.g. hallucinations) were more prominent than negative symptoms (e.g. affective blunting). Almost half the participants (43%) had previous episodes of methamphetamine-induced psychosis. Within this group, all had defaulted on the prescribed treatment prior to admission. Only 29% of the participants had received prior formal substance-use rehabilitation as treatment for their disorder. High rates of comorbid cannabis and alcohol use (51%) were recorded. Most of the participants required transfer to specialist psychiatric hospitals. The amounts of methamphetamine used were not a predictor of the persistence of psychosis; however, the pattern of use was.

**Conclusion:**

Clinical features correspond with other international findings. The currently employed model of sequential, non-integrated psychiatric and substance use treatment in this setting appears ineffective.

## Introduction

Methamphetamine abuse is a global problem, with the Western Cape, South Africa, having emerged as a region with one of the highest levels of its use.^1^ At 33%, crystal methamphetamine is the most common primary drug of abuse for patients presenting to specialist substance abuse treatment centres.^2^ Methamphetamine-related psychiatric problems, especially methamphetamine-induced psychosis, places a significant burden on psychiatric inpatient services.^3^ Despite this increase in admissions for methamphetamine-induced psychosis, there have been no local studies examining the clinical characteristics, treatment modalities or outcomes of patients admitted with methamphetamine-induced psychosis.

Methamphetamine is often consumed several times over the course of many days, in runs or binges by users. Routes of administration include intranasal sniffing, inhalation, intravenous use and oral ingestion. The preferred method of methamphetamine use varies by geographical region and has changed over time.^4^ In South Africa, methamphetamine is generally smoked in a glass pipe called a ‘lolly’ or ‘pop-eye’, or using a straw and the globe of a light bulb (Weich L., 2015, Personal communication). Smoking methamphetamine leads to a very fast uptake of this lipophilic drug in the brain, amplifying methamphetamine’s addiction potential.^5^ Adverse health consequences include both medical and psychiatric symptoms, such as anxiety, depression, suicidal ideation, aggression and psychosis.^6^ The potential for methamphetamine to induce a psychotic state is well established and is considered one of the most serious outcomes of use.^7,8^

## Methamphetamine-induced psychosis

Methamphetamine-induced psychosis is typically transitory in nature, with psychotic symptoms usually abating within days following cessation of methamphetamine use.^9^ However, it has been suggested that about 5% – 15% of users experience persistent psychosis despite abstinence.^10,11^ Not all methamphetamine users experience psychotic symptoms. Current identified risk factors include heavier use, premorbid schizoid and schizotypal personality traits, family loading of psychotic disorders, other substance use, sexual abuse and previous head-injury or neurological disorder.^12,13^ International studies have not reported an association between sociodemographic factors or method of use and the appearance of methamphetamine-induced psychosis.^7^

While a single dose of methamphetamine is able to induce a transient psychotic episode, heavy use of methamphetamine is associated with an increased likelihood of experiencing psychosis. There is also some indication that high-dose binge use of methamphetamine often precedes the onset of psychotic symptoms. McKetin et al. found that among long-term methamphetamine users, there was a five-fold increased likelihood of psychotic symptoms during periods of methamphetamine use compared with periods of abstinence. The risk was dose-dependent and further doubled by frequent marijuana or alcohol use.^7^

### Clinical features

Clinically, there are striking similarities between methamphetamine-induced psychosis and the psychotic symptoms associated with schizophrenia.^12^ These similarities may lead to diagnostic uncertainty, especially in the acute setting. A number of studies have compared the clinical presentation of methamphetamine-induced psychosis with that of schizophrenia. Some studies have concluded that methamphetamine-associated psychosis can be distinguished from schizophrenia by the absence of thought disorders (tangential thought, derailment and neologisms) and the presence of less negative symptoms (poverty of speech, psychomotor retardation, flattened affect and loss of drive).^8^ Delusions have typically been described as persecutory in nature, while hallucinations are most often auditory or visual, but can occur in any of the other senses (e.g. olfactory).^8,12,13,14^

### Treatment modalities

The treatment of methamphetamine-induced psychosis in acute settings usually involves the administration of benzodiazepines with or without an antipsychotic agent to sedate the patient and alleviate psychotic symptoms. Given the time-limited nature of symptoms, benzodiazepines are often sufficient; there is no consensus among clinicians regarding preference of agent. The paucity of research in this area is highlighted by a 2008 Cochrane Review that found only one randomised controlled trial on treatment of amphetamine-induced psychosis.^15^ Since then, only single case reports^16^ and one other double-blind randomised controlled trial^17^ have been added to the published evidence base. Developing setting-specific treatment guidelines for the management of behavioural disturbance could potentially reduce the risk of harm to both the patient and health professionals. This however requires knowledge of current acute treatment procedures.

Although substantial literature has been accumulated from other countries,^8,11,14^ no South African study has specifically described the clinical features, treatment or outcomes of patients admitted to acute psychiatric services with methamphetamine-induced psychosis. The examination of the clinical features of substance-induced psychotic disorders in the acute setting is important as it can potentially identify differentiating clinical features independent from the effects of illness course and chronic treatment. Ultimately, such information can improve diagnostic efficiency and serve as a foundation to develop targeted clinical interventions and guide appropriate mental health service planning.

### Aims

This pilot study aimed to address specific questions regarding the nature of psychotic symptoms, outcomes and treatment modalities currently utilised among patients presenting with methamphetamine-induced psychosis to inpatient psychiatric services in the Western Cape, South Africa.

## Methodology

### Design and setting

This was a pilot, cross-sectional descriptive study conducted at three district level Western Cape Government hospitals from 1 March 2014 to 31 August 2014, namely Helderberg, Karl Bremer and Eersterivier Hospitals. These hospitals have been identified as designated centres by the South African Mental Health Care Act to admit, observe and treat mental health care patients for 72 hours prior to admission to a psychiatric hospital. In most cases, psychotic symptoms typically resolve prior to completion of the 72-hour observation period and thus transfer to tertiary psychiatric hospitals is often not required.

### Participants

All patients, aged between 18 and 65 years, admitted to inpatient services with psychotic features associated with methamphetamine use were eligible for inclusion in this study. Recent methamphetamine use was determined by self-report, collateral obtained from informant and confirmed by urine toxicology. Diagnosis of methamphetamine-induced psychosis was based on the accepted signs and symptoms of a clinical presentation according to the Diagnostic and Statistical Manual (DSM-V) criteria for substance-induced psychotic disorder.^18^ Any previous diagnosis of a non-substance-associated psychotic disorder (e.g. schizophrenia, schizoaffective disorder) was exclusionary.

### Procedure

Ethics approval to conduct this study was obtained from the Human Research Ethics Committee (HREC), Stellenbosch University (Nr S14/01/010), as well as from the provincial government of the Western Cape and individual medical superintendents/managers of the respective hospitals. This study was conducted in accordance with the International Committee for Harmonization Good Clinical Practice (GCP) guidelines and South African GCP.^19^

### Recruitment and consent

Only patients that were considered clinically stable enough by the treating clinicians were approached for participation. Potential participants were given an explanation of the nature, purpose and procedures of the study and an information sheet was provided in the participant’s home language. All patients who met eligibility criteria and provided written informed consent had a structured face-to-face interview conducted by the primary investigator in a private interview room within 1–7 days of admission. A history of a chronic psychotic disorder was assessed by asking participants whether they have ever been told by a doctor that they may have schizophrenia, schizoaffective disorder, schizophreniform or bipolar affective disorder, as well as collateral obtained from family/clinical notes. Each interview took between 45 and 60 minutes. Confidentiality and anonymity of participants were maintained by not using names of study participants or any other form of identification in the research instruments. Although specific identifying data (such as full name and date of birth) were collected for tracking and study surveillance, all participants were given a unique identification (ID) number. All clinical information was maintained in a secure, password-protected electronic database system.

### Clinical and functional measures

A structured data sheet was compiled for the purpose of this study. Pertinent sociodemographic and clinical data collected included psychiatric diagnostic and treatment history and family psychiatric and substance use history. The duration of hospitalisation prior to discharge or transfer to the next level of care was recorded. The type, dosage and frequency of all medications administered were captured.

### Measure of psychopathology

The 24-item Brief Psychiatric Rating Scale (BPRS), version 4.0, was used to measure the presence and severity of current psychiatric symptoms.^20^ Symptoms measured by the BPRS include somatic concerns, anxiety, depression, suicidal ideation, guilt, hostility, elevated mood, grandiosity, suspiciousness, hallucinations, unusual thought content, bizarre behaviour, self-neglect, disorientation, conceptual disorganisation, blunted affect, emotional withdrawal, motor retardation, tension, uncooperativeness, excitement, distractibility, motor hyperactivity, mannerisms and posturing.

The presence and severity of psychiatric symptoms are rated on a Likert scale ranging from 1 (not present) to 7 (extremely severe). Possible scores vary from 24 to 168, with lower scores indicating less severe psychopathology. Suggested cut-off scores are: 0–30 denoting no notable illness, 31–40 minimal illness, 41–53 moderate illness and 53 + marked illness.^21^ The BPRS has been shown to be effective in various substance-use populations.^17^ The BPRS has also been used in various research studies in which the relationship between major psychiatric illness and substance misuse has been investigated.^22^

### Substance use

The route, frequency and pattern of nine classes of substance use (methamphetamine, alcohol, cannabis, solvents, Mandrax, hallucinogens, heroin, benzodiazepines and others specified) were assessed using a 3-month time-line follow back.^23^ To address the variations in literacy of participants, all questions were read aloud and recorded by the interviewer.

### Data analysis

Data analyses were performed using Statistica software version 11 (STATISTICA data analysis software system, Statsoft Inc. 2012, www.statsoft.com). Descriptive statistics were reported as means and standard deviations for ordinal data (e.g. age and length of stay) and frequencies as percentages of diagnoses. One-way ANOVA’s were used to compare ordinal data between groups, while cross-tabulations with the Chi-square test was used to compare categorical variables. Spearman correlations were used to test for associations between ordinal variables. Significance was set at *p* < 0.05.

## Results

### Sample

Over a 6-month period, 58 inpatients met study criteria and were invited to participate. Fifty-six individuals consented, while two patients declined, one owing to suspicion and the other owing to lack of interest. The average time from admission to interview was 3 days.

### Demographic data

Participants were predominantly male (*N* = 43; 77%), single (*N* = 50; 89%), unemployed (*N* = 40; 71%) and most (*N* = 50; 89%) had completed some secondary level of education.

### Past psychiatric history

While 32 (57%) were index psychiatric admissions, 24 (43%) of the participants had been admitted for previous episodes of methamphetamine-induced psychosis, with an average of 1.9 episodes. Within this group, 100% had defaulted prescribed treatment prior to admission (haloperidol = 5, risperidone = 16, chlorpromazine = 1, clopixol depot = 1 and fluanxol depot = 1). Thirty-six (64%) had a positive family history of psychiatric illness, of which the majority of cases (40%) were that of mood-disorders.

### Substance use

The mean (SD) debut age of any substance use was 17 (SD 6. 2) years, of which the majority was cannabis (64%). The mean (SD) debut age of methamphetamine use was 20 (SD 7.5) years. The majority (72%) reported last use more than 24 hours prior to admission.

Twenty-four (43%) of the participants endorsed daily use of 1–2 lollies, by inhalation, 19 (34%) reported a weekend-binge pattern of use and the remaining 23% reported an inconsistent pattern of use. Significantly, 29 (51%) participants had frequently used other substances in the past 3 months. Most notable were the use of cannabis and alcohol. Twenty-six (46%) had positive urine screening tests for other substance use with the current admission. Cannabis was the most frequently reported substance of abuse (92%).

Forty four (79%) of the participants reported a positive family history of substance use, mostly for methamphetamine, cannabis and alcohol.

Only 16 (29%) of the participants had received prior formal treatment for their substance use disorder. Thirty-three (59%) participants reported previous arrests and incarcerations, of which 95% were related to their substance use disorder.

### Admission and outcome data

Most participants (34; 61%) were police-accompanied on admission. The mean (SD) length of stay of all participants in this study was 7.4 (SD 4.3) days. Twenty-nine (52%) of the participants were to be transferred to specialist psychiatric hospitals owing to persistent psychotic symptoms. The clinical decision to refer to a specialist psychiatric hospital was made following the 72-hour MHCA observation period. Although the amounts of methamphetamine use (as measured by the number of units/‘lollies’ used over the past 3-month period) did not predict the persistence of psychosis, those who had endorsed a daily pattern of use were more likely to be transferred to the next level of care.

The most commonly employed treatment agent was the antipsychotic risperidone (46; 82%), at an average dose of 1 mg once daily, and benzodiazepine and lorazepam (50; 89%) in a *pro re nata* (prn) dosing schedule.

### Clinical profile

**FIGURE 1:**
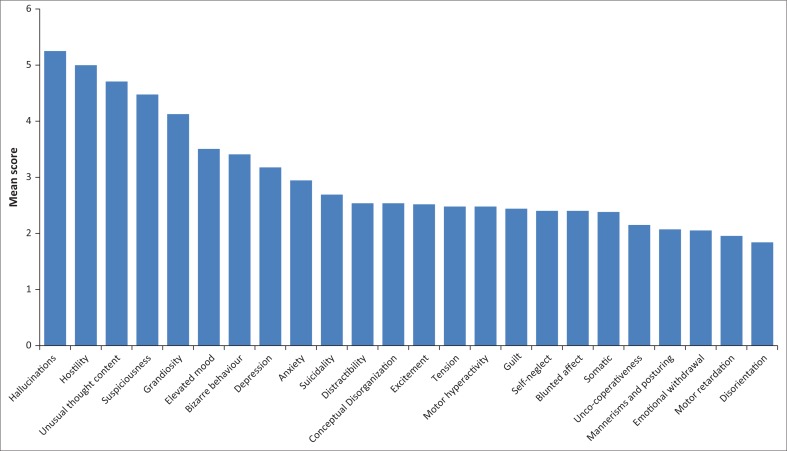
Bar plot of the mean Brief Psychiatric Rating Scale scores.

An average of 3 hours of sleep per night in the week prior to admission was reported. The mean BPRS score was 69, with a difference present in measured severity between the genders (males = 73, females = 66). The highest overall scores were obtained in the hallucination, hostility, unusual thought content, suspiciousness and grandiosity subscales (Figure 1). Few participants were assessed as currently exhibiting self-neglect, blunted affect or emotional withdrawal. A higher BPRS score predicted the persistence of psychotic symptoms as well as transfer to specialist psychiatric hospitals.

## Discussion

The overall impetus for this pilot study came from the increasing prevalence and resulting public health problem posed by illicit methamphetamine use. Although clinical research is required for the development of methamphetamine-induced psychosis services, few local studies have been carried out.

Similar to other studies on methamphetamine-induced psychosis, participants in this study were mostly male, single and unemployed.^8,14,24^ Interestingly, most had obtained some level of secondary schooling, which differs from other international findings where the majority of participants had either limited schooling or were illiterate.^8,14^ Similar to international findings, high rates of concomitant use of other substances was recorded.^8,14^ The most common patterns of use were either daily or weekend-binge patterns. All participants had used methamphetamine via inhalation. This is the first description of patterns of use in a South African clinical setting.

In this study, known risk factors from international studies were also demonstrated among participants. Specifically, sleep deprivation,^25^ concomitant use of other substances^8^ and high dose – binge-pattern use.^7^ Potentially, the emergence of psychosis in this sample may also be related to differences in the type of methamphetamine-induced exposure or patterns of use. Locally produced methamphetamine may differ in composition from methamphetamine synthesised in other areas of the world. This may result from differences in both the manufacturing process as well as the by-products used. In addition, poly-drug use is common in South Africa with unique drug combinations such as methaqualone and methamphetamine.

Of note was the high number (24; 43%) of patients who had previous episodes of methamphetamine-induced psychosis. Japanese literature has noted that methamphetamine users may display a rapid recurrence of psychotic symptoms on re-exposure to the drug.

The average BPRS score of 69 suggests marked illness according to the suggested cut-off score of 53 + by Leucht et al. in 2005.^21^ Of note is the clinical profile of more positive psychotic symptoms (e.g. hallucinations) versus negative symptoms (e.g. affective blunting) which is in keeping with other international studies.^8^ If these findings could be replicated on a larger scale, it may assist clinicians in their differential diagnostic formulations.

Of concern were the high rates of non-adherence (100%) to current prescribed treatment regimens and low rates of formal substance use disorder treatment attempts (16; 29%). One possible reason for this non-adherence could be that the currently employed model of sequential, non-integrated psychiatric and substance use services is ineffective in the cohort studied. In South Africa, the funding and responsibility for substance use and mental health programmes is divided between the departments of social development and health. During admission periods, patients are referred to a social worker who provides information regarding substance treatment options. Following discharge, it is left to the patient to navigate these disjointed services. Poor uptake of substance treatment may be owing to a number of reasons such as social or environmental barriers, lack of readiness to change, ongoing psychotic symptoms and long waiting lists at state drug-rehabilitation centres.

Most of the participants in this study required transfer to specialist psychiatry facilities as their psychotic symptoms persisted. This further highlights the burden that methamphetamine use is placing on mental healthcare services in South Africa and the need for addiction-psychiatry to be prioritised in health reforms and bills.

### Strengths and limitations

A well-known and established rating scale^20,22^ was employed during clinical interviews, and care was taken to distinguish between substance-induced and non-substance-induced psychosis. However, this pilot study had a relatively small sample size which limits the power of the study. In addition, most patients were receiving psychotropic medications at the time of their participation, and this may have had an impact on the findings, particularly the scores on the BPRS. The possible effect of substance intoxication on symptom rating at initial presentation (i.e. within 1–2 days of hospital admission) is important as is the timing of clinical examination. Finally, as many participants were using other substances, mostly cannabis with this admission episode, it is not possible to determine the specificity of the findings to methamphetamine use.

### Recommendations

Differentiating methamphetamine-induced psychosis from a primary psychotic disorder, such as schizophrenia, may be challenging. Diagnostic accuracy may be improved by employing objective indicators of recent use such as urine toxicology, as well as assessing the temporal relationship of symptom onset to methamphetamine use.

An episode of methamphetamine-induced psychosis should not be regarded as a singular episode. The findings of this study suggest that a prior episode of methamphetamine-induced psychosis confers risk for further episodes. Patient diagnosed with this disorder should receive ongoing treatment, follow-up and care. Psychosocial treatment for methamphetamine-use disorders have a strong evidence base and is considered the first-line treatment to reduce rates of psychosis by preventing relapse.^7^ We recommend the adoption of an integrated, co-ordinated mental health and substance use service. Patients should ideally receive treatment for both conditions from the same clinical team during the admission period. This will require acquisition of necessary skills by mental health care practitioners.

Many questions regarding the clinical features and the best treatment choice for methamphetamine-psychosis remain unanswered. Although this was a small self-funded pilot study, the investigators hope that this study may be replicated and expanded upon in other provinces to inform and optimise current treatment models.

## Conclusion

Clinically, the most prominent features were positive psychotic symptoms rather than negative symptoms, which is in keeping with related international findings. Further salient findings in this cross-sectional descriptive study include a high number of patients who had prior documented episodes of methamphetamine-psychosis, as well as a 100% non-adherence rate to prescribed treatment regimens. The currently employed model of sequential (non-integrated) psychiatric treatment and substance treatment services appears ineffective. Improved outcomes will require implementation of timely and comprehensive standard interventions.
